# Case report: A case of laterally spreading duodenal cancer with slight submucosal invasion accompanied with concurrent lymph nodes metastasis

**DOI:** 10.1002/deo2.100

**Published:** 2022-02-26

**Authors:** Kentaro Iwata, Motohiko Kato, Atsushi Nakayama, Takanori Kanai, Naohisa Yahagi

**Affiliations:** ^1^ Department of Internal Medicine Division of Gastroenterology and Hepatology Keio University School of Medicine Tokyo Japan; ^2^ Division of Research and Development for Minimally Invasive Treatment Cancer Center Keio University School of Medicine Tokyo Japan

**Keywords:** duodenal cancer, endoscopic submucosal dissection

## Abstract

A 70‐year‐old female diagnosed with duodenal cancer was referred to our hospital. Esophagogastroduodenoscopy revealed an 80 mm flat elevated lesion was located in the inner wall of the second part of the duodenum and the lesion completely involved major papilla. Endoscopic submucosal dissection (ESD) was performed and the lesion was resected in a single piece including the part of the major papilla. The pathological examination of the resected specimen showed moderately differentiated adenocarcinoma limited in the mucosa in most parts of the lesion, however, cancer cells invaded into the submucosal layer with an invasion depth of 100 μm in only a small area. Lymph ductal involvement was confirmed in that area. Two months after ESD, pylorus‐preserving pancreatoduodenectomy combined with extended lymph node dissection was additionally performed. The postoperative pathological examination revealed lymph ductal involvement was observed in the regional lymph node. While the postoperative clinical course was uneventful, systematic metastasis was pointed out 5 months after surgery. The patient was died 9 months after surgery. Due to its rarity, the natural history of duodenal cancer has been still unclear. In this case, even a lesion with only a localized small area of submucosal invasion developed systemic metastasis, indicating the high malignant potential of duodenal cancer.

## INTRODUCTION

Duodenal cancer is relatively rare and its biological behavior has not been fully understood yet. Here, we report a case of large laterally spreading duodenal cancer with invasion into the submucosa. In this case, invasion depth was limited only to slight submucosa, however, the patient presented lymph ductal involvement and developed systemic metastasis relatively early after radical surgery.

## CASE REPORT

**FIGURE 1:**
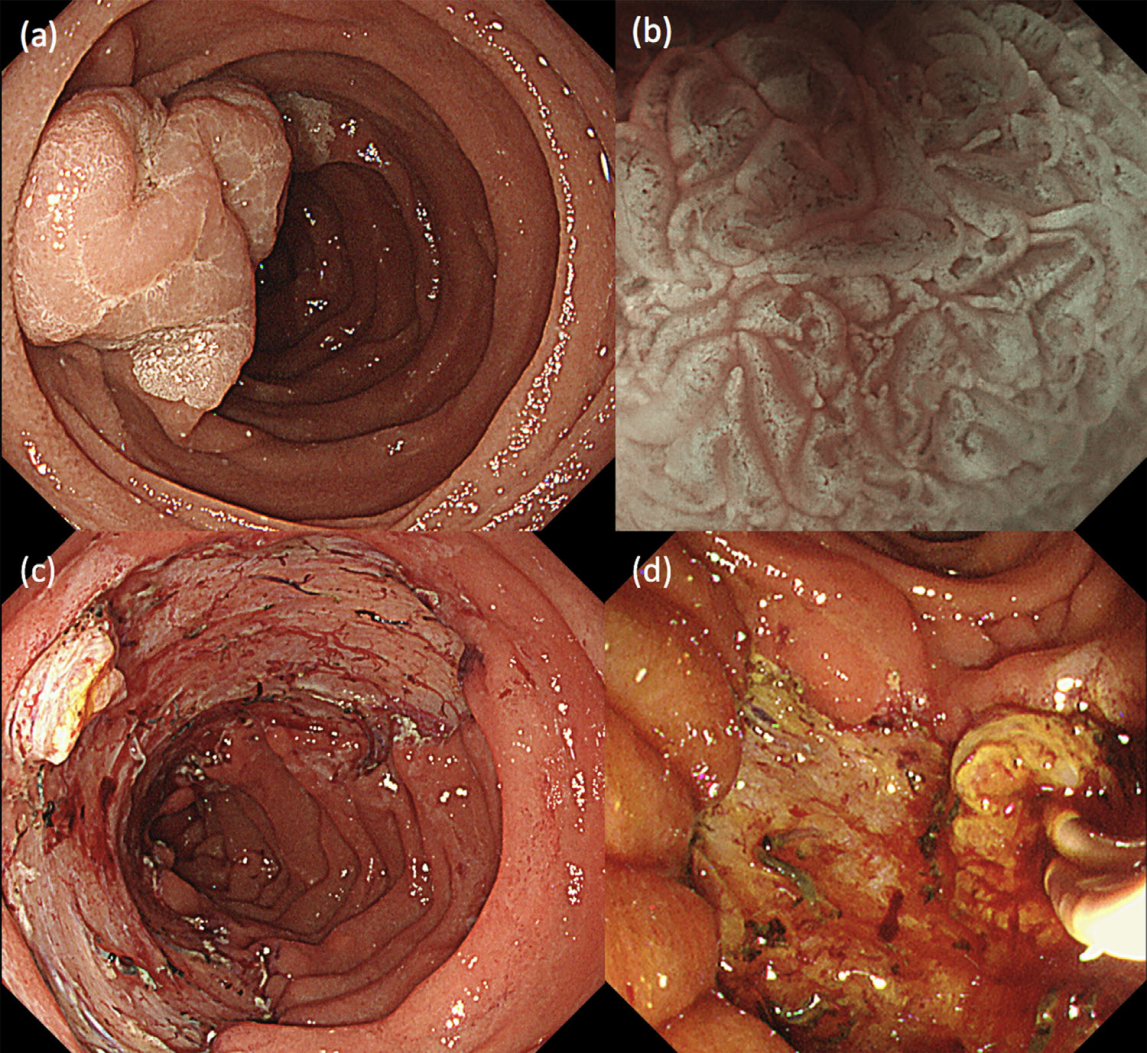
(a) The duodenal tumor was presented at the inner part of the descending duodenum to the inferior duodenal angle. (b) With magnified image‐enhanced endoscopy, the surface structure was evenly well‐organized but microvessels could not be detected, since white opaque substances were visualized on the entire surface of the tumor. (c) The resected ulcer was approximately half‐circle of the lumen including a major papilla. (d) Endoscopic naso‐biliary and naso‐pancreatic drainage (ENBPD) tubes were inserted to prevent delayed adverse events due to exposure of bile and pancreatic juice to the resected wounds

**FIGURE 2:**
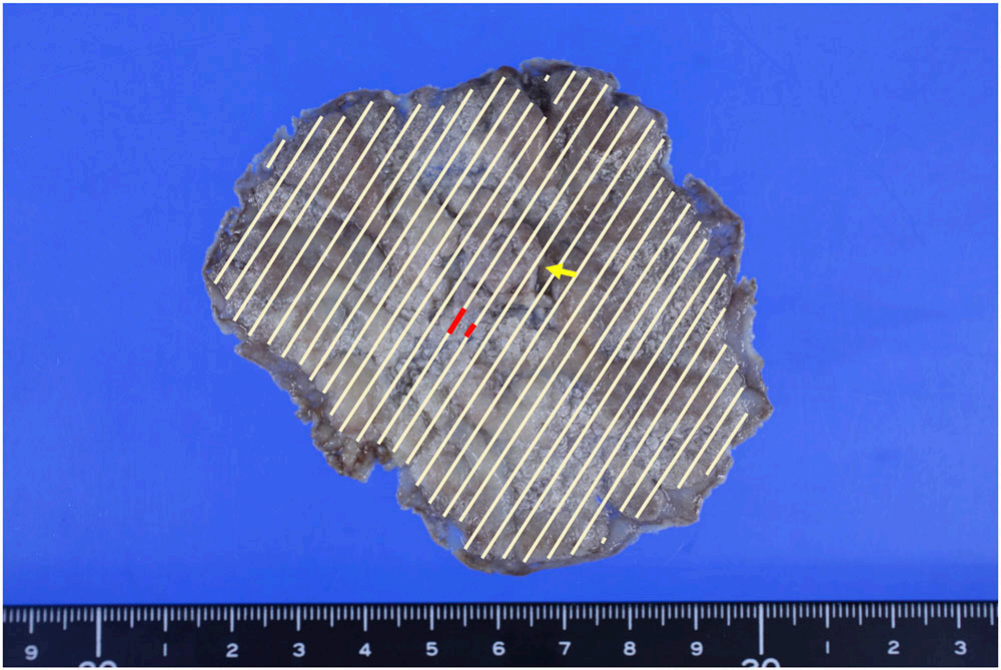
The mapping image of postoperative pathology. The horizontal margins were totally negative and the submucosal invasion was localized to a small area near the major papilla (beige line: intraepithelial tumors, red line: submucosal invasive cancer, yellow arrow: major papilla)

**FIGURE 3:**
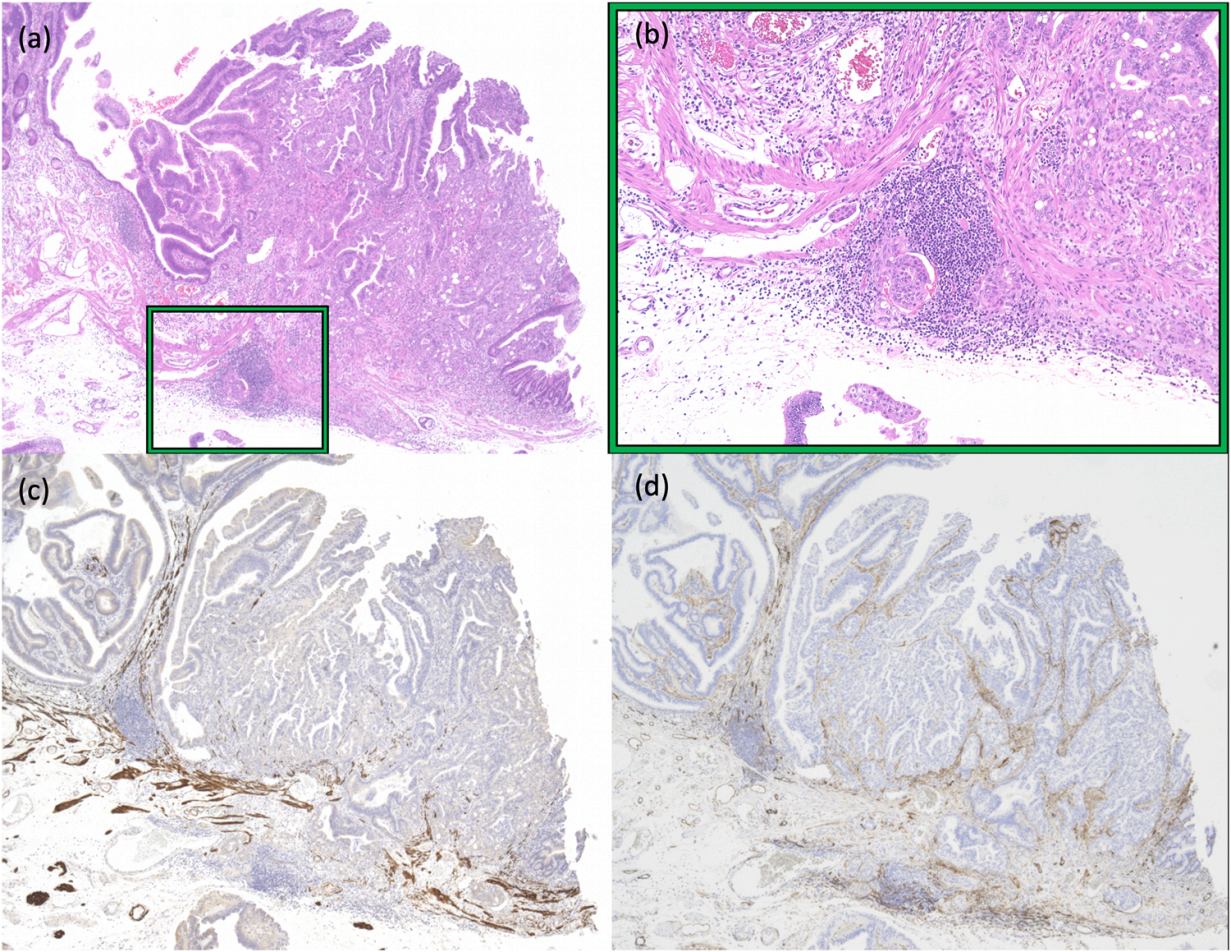
Immunohistological staining for hematoxylin and eosin (H‐E) staining (a) 20x, (b) 100x, (c) desmin staining, and (d) D2‐40 staining. The muscularis mucosa was disrupted by invading cancerous cells (approximately 100 μm) and they were presented in several lymph ducts. The final pathological diagnosis was moderately differentiated adenocarcinoma with tubular adenoma (intestinal type), invasion of 100 μm into the submucosal layer, INFb, ly1, v0, pHM0, pVM1, and budding grade 2

**FIGURE 4:**
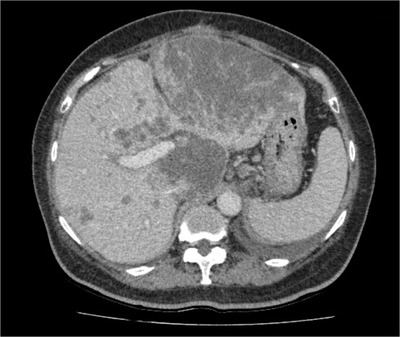
Six months after pylorus‐preserving pancreatoduodenectomy combined with extended lymph node dissection, the patient complained of abdominal pain and bloating. Computer tomography (CT) scans showed systemically metastasized to liver and peritoneum

A 70‐year‐old female patient was referred to our hospital for the treatment of a large laterally spreading duodenal tumor. Esophagogastroduodenoscopy (EGD) for preoperative evaluation revealed the tumor was located at the inner part of the descending duodenum to the inferior duodenal angle (Figure 1a). The tumor was over 80 mm in size with the extension of more than 50% of the duodenal lumen. With magnified image‐enhanced endoscopy, the surface structure was evenly well‐organized but microvessels could not be detected, since white opaque substances were visualized on the entire surface of the tumor (Figure 1b). Major papilla was completely involved by the tumor with the side‐viewing endoscope and bile duct invasion did not present with endoscopic ultrasonography. We diagnosed the possibility of early duodenal cancer based on the size of the lesion, but there was no sign of deep submucosal invasion. We planned endoscopic submucosal dissection (ESD) under general anesthesia to resect the lesion in a single piece. The procedure was performed by using GIF‐H290T (Olympus, Japan) attached the ST hood (Fujifilm medical, Japan) to the tip. First, we made an incision at the anal side that was poor visibility and maneuverability. We added a further incision at the bilateral side, then we carefully dissected the submucosal layer from the oral side with a water pressure method.^1^ After dissecting all areas except the major papilla, it was dissected carefully and en bloc resection was achieved without any adverse event (Figure 1c). Finally, we inserted an endoscopic naso‐biliary and naso‐pancreatic drainage (ENBPD) tubes to prevent delayed adverse events due to exposure of bile and pancreatic juice to the resected wounds (Figure 1d). Her postoperative course was stable with no complications. ENBPD was removed 4 days after ESD and food intake was started. She was discharged 9 days after ESD. Histopathological examination revealed that the large part of the lesion was intra‐mucosal moderately differentiated adenocarcinoma, but cancer cells invaded slightly into the submucosal layer (100 μm) in only a small area close to the major papilla (Figure 2). Lymph ductal involvement was observed within the area of submucosal invasion with D2‐40 staining. As for resection margin, the free horizontal margin was pathologically confirmed, however in the part of the major papilla, adenomatous cells extended along the bile duct and vertical margins were diagnosed as positive, even though no cancerous component was observed. In addition, immunohistological staining for MUC2 and CD10 was positive, while MUC5 and MUC6 were both negative, therefore the tumor mucin phenotype was estimated intestinal type. The final pathological diagnosis was moderately differentiated adenocarcinoma with tubular adenoma (intestinal type), invasion of 100 μm into the submucosal layer, INFb, ly1, v0, pHM0, pVM1, and budding grade 2 (Figures 3a–d). We referred her to the surgeon for additional surgery. Two months after ESD, pylorus‐preserving pancreatoduodenectomy combined with extended lymph node dissection was performed. The postoperative pathological diagnosis revealed residual tubular adenoma with severe atypia in the major papilla region of post‐ESD scar and metastasis of adenocarcinoma in the lymph nodes attached to the duodenum. He was doing well for a while after the surgery. However, 5 months after surgery, abdominal pain and bloating gradually appeared. Abdominal CT scans showed multiple liver masses, ascites, and multiple enlarged lymph nodes (Figure 4). She was diagnosed as clinical stage IV of postoperative duodenal cancer. Chemotherapy was immediately considered but her physical condition was gradually worsened. Finally, the patient died 9 months after surgery.

## DISCUSSION

With the spread of endoscopy for health care, the detection number of duodenal tumors has been increasing in recent years. Although the natural history of duodenal tumors is still unclear, the poor prognosis of duodenal cancer has been gradually recognized. In this case, only a localized small area of submucosal and lymph ducts invasion eventually led to systemic metastasis, and this case symbolically indicates the high biological malignancy of duodenal cancer. Recently, Yoshimizu et al. reported lymph nodes metastasis was presented in 46% of duodenal cancer with submucosal invasion.^2^ Our case report is in line with their report that submucosal invasive duodenal cancer has an increased risk of lymph ductal involvement and lymph nodes metastasis. Moreover, in our case, invasion depth was only 100 μm in the limited area of a large lesion, and we should be aware of the high malignant potential of duodenal cancer.

Most duodenal epithelial tumors are low‐grade adenoma without invasion and metastatic capability, and it is essential to differentiate the rare lesions with aggressive biological phenotype from these majority low‐grade lesions. In the gross appearance of superficial duodenal tumors, type 0‐Ⅰ and 0‐Ⅱc account for 20%, and type 0‐IIa accounts for 60% of the total.^3^ Noteworthy, there is no significant difference in the proportion of gross type between low‐grade dysplasia and high‐grade dysplasia or non‐invasive superficial cancer.^3^ Therefore, although depressed lesions are generally considered high malignancy in the colon, morphological evaluation is insufficient to diagnose malignant potential in the duodenum. The duodenal tumor results in a laterally spreading tumor‐like morphology by developing laterally along the lumen. In contrast, unlike colorectal tumors, the sessile serrated lesion does not exist in the duodenum. Some recent studies reported magnifying endoscopy with image enhanced endoscopy (IEE‐ME) could predict histological grade.^4^ Nakayama et al. reported open‐loop type micro‐surface structure and absence of white opaque substance by IEE‐ME are independent predictors for high‐grade histology of resected specimen as well as tumor size.^5^ Furthermore, duodenal tumors are classified into gastric, intestinal, and mixed types according to mucin phenotype. The gastric type is classified by immunostaining for MUC5AC and MUC6, while the intestinal type is classified by MUC2, CD10, and CDX‐2. Moreover, lesions in the proximal duodenum have a higher gastric type frequency. In contrast, those in the distal duodenum have a higher frequency of intestinal‐type. The former is a more biologically malignant potential than the latter.^6^ Based on these factors, the endoscopic diagnosis of duodenal tumors requires a comprehensive judgment from various findings. In the present case, because the tumor diameter was large, but the morphology and surface structure were well organized, we finally diagnosed low‐grade dysplasia or cancer that remains in the mucosa at worst.

The guideline on superficial duodenal epithelial tumors published by the European Society for Endoscopy mentions that duodenal ESD needs to be considered carefully due to its high incidence of complications,^7^ and endoscopic mucosal resection with piecemeal resection may be acceptable as a treatment option for lesions with seemingly difficult of ESD. However, as shown in this case, even if the endoscopic diagnosis was low‐grade dysplasia, the result can be invasive cancer. Furthermore, it is extremely difficult to make an accurate pathological diagnosis for only 100 μm submucosal invasion or lymph ductal involvement especially in specimens obtained by piecemeal resection. Therefore, it is necessary to aim for en bloc resection unless it is definitely diagnosed as a low‐grade adenoma. For more reliable en bloc resection, ESD or surgery is a choice. However, whichever treatment was chosen, these are extremely invasive and high risk for complications.^8^, ^9^, ^10^ Recently, modified ESD techniques including the water pressure method and prophylactic means such as the string clip suturing method are reported.^1^ Moreover, as minimally invasive surgical treatment options, duodenal laparoscopic and endoscopic cooperative surgery have made it practically achievable to perform secure en bloc resection of duodenal cancer.^11^ We should determine appropriate treatment strategies depending on the patient's condition and the character of the lesions.

In conclusion, this case suggests duodenal cancer has an extremely high biological malignancy once it invades the submucosal layer, and en bloc resection should be considered for accurate pathological examination if high‐grade histology is predicted based on endoscopic findings.

## CONFLICT OF INTEREST

The authors declare no conflicts of interest.

## ETHICS STATEMENT

Not applicable.

## FUNDING INFORMATION

None.
